# Supplementary material for: Comparison of Long COVID-19 Caused by Different SARS-CoV-2 Strains: A Systematic Review and Meta-Analysis
Source: Int J Environ Res Public Health. 2022 Nov 30;19(23):16010. doi: 10.3390/ijerph192316010 (PMC9736973; doi:10.3390/ijerph192316010)
Supplement: Supplementary file 1 [file ijerph-19-16010-s001.zip › ijerph-1961520-supplementary.pdf]

Table S1 Included Studies

Author	Sample size	Main virus strains	Newcastle–Ottawa quality assessment scale	Countries
Ahmet Naci Emecen[1]	5083	Omicron	low	Turkey
Aleksander Och[2]	79	Omicron	low	Poland
Anna Lindahl[3]	101	Wild	low	Finland
Beatriz CostaTodt[4]	251	Wild	low	Brazil
Christoph Becker[5]	90	Wild	low	Switzerland
Dai S[6]	50	Wild	moderate	China
Damiano Caruso[7]	118	Wild	low	Italy
Daniel CruzBretasaArnaldo[8]	211	Gamma	low	Brazil
Daniele Noviello[9]	164	Wild	low	Italy
Danilo Buonsenso[10]	245	Omicron	low	Italy
David Zuschlag[11]	162	Delta	low	Germany
Dominik Menges[12]	431	Wild	low	Switzerland
Elena Tessitore[13]	184	Wild	low	Switzerland
Fangyuan Zhou[14]	120	Wild	low	China
Florian Desgranges[15]	418	Wild	moderate	Switzerland
Förster[16]	1459	Wild	moderate	Germany
G Shiny Chrism Queen Nesan[17]	1354	Delta	low	India
Gabriele da Silveira Prestes[18]	44	Wild	low	Brazil
GeorgeCalcaianua[19]	320	Wild	low	France
Gianna Vargas Centanaro[20]	305	Wild	low	Spain
Gonzalo Labarca[21]	60	Wild	low	Chile
Hadar Klein[22]	103	Wild	moderate	Israel
Ignacio Martin-Loeches[23]	991	Wild	low	Spain
Jessica González[24]	62	Wild	moderate	Spain
Judit Aranda[25]	113	Wild	low	Spain
Jun Zhang[26]	248	Wild	low	China
Justin R. Kingery[27]	530	Wild	low	United States of America
K Zhan[28]	311	Wild	low	China
KWMiskowiak[29]	25	Wild	low	Denmark
LouiseSigfrid[30]	327	Alpha	low	United Kingdom
Maddalena Peghin[31]	599	Wild	low	Italy
Marco Colizzi[32]	479	Wild	low	Italy
Marie Vejen[33]	128	Alpha	moderate	Denmark
Marina Aiello[34]	121	Delta	low	Italy
Mario Rivera-Izquierdo[35]	453	Wild	low	Spain
Marta Rigoni[36]	471	Wild	low	Italy

Martin Mølhave[37]	45	Wild	low	Denmark
Miriam Hernández Porto[38]	72	Wild	low	Spain
Mohamed Attauabi[39]	222	Omicron	low	Denmark
Mohammad Anwar Hossain[40]	2198	Beta	low	Bangladesh
Philippine ELOY[41]	324	Alpha	low	France
Priya Duggal[42]	328	Wild	low	United States of America
Ramos-Sánchez Mónica[43]	305	Wild	low	Spain
Raphael S. Peter[44]	11710	Omicron	low	Germany
Rebecca C. Robey[45]	221	Wild	low	United Kingdom
Ricardo Titze-de-Almeida[46]	236	Wild	low	Brazil
Souheil Zayet[47]	354	Wild	low	France
Xuejiao Liao[48]	273	Wild	low	China
Yumin Li[49]	141	Wild	low	China
Álvaro Aparisi[50]	70	Wild	low	Spain
Álvaro Romero-Duarte[51]	797	Wild	low	Spain

Table S2. Pooled prevalence of COVID-19 consequences at follow-up 3 months and above

Consequences	Number of Studies	Patients n/N	PP (95%CI, %)	p-value	I2	p-value for Egger regression test
**General symptoms**						
≥1 Symptoms	24	5121/12870	51.1 (41.4, 60.8)	<0.05	99.2%	0.2394
Fever or Feverishness	13	289/15540	2.7 (1.9, 3.6)	<0.05	92.3%	<0.0001
Fatigue	26	5520/25062	29.8 (24.4, 35.1)	<0.05	99.1%	0.01
Muscle weakness	6	142/3329	4.9 (2.8, 7.0)	<0.05	96.5%	<0.0001
Myalgia	16	1724/15944	10.9 (7.6, 14.3)	<0.05	98.5%	0.0008
Joint pain or arthralgia	9	2290/12635	18.5 (13.5, 23.5)	<0.05	93.7%	0.0718
Headache	28	3985/27799	10.6 (7.8, 13.3)	<0.05	99.4%	0.0001
Dizziness or Vertigo	10	2199/9334	3.5 (1.8, 5.2)	<0.05	96.2%	<0.0001
Olfactory abnormalities	8	2697/16896	9.1 (0.7, 17.5)	<0.05	99.6%	0.6648
Olfactory loss	11	503/5301	13.2 (9.1, 17.3)	<0.05	97.1%	0.006
Taste abnormalities	7	2070/16793	8.3 (0.9, 15.6)	<0.05	99.6%	0.3641
Taste loss	11	445/7590	8.9 (6.1, 11.6)	<0.05	97.4%	<0.0001
Hair loss	9	1781/14037	9.6 (4.7, 14.4)	<0.05	98.6%	0.0204
Cutaneous or Skin disorders	6	156/3738	3.9 (2.1, 5.7)	<0.05	88.3%	0.3123
Rash	5	666/16548	3.3 (0.4, 6.1)	<0.05	99.3%	0.2735
**Respiratory symptoms**						
Cough	27	2447/24229	13.3 (10.6, 16.0)	<0.05	98.8%	<0.0001
Gamma	1	34/156	21.8 (15.3, 28.3)	<0.05	-	
Dyspnea	27	2105/16467	21.2 (17.7, 24.7)	<0.05	99.1%	<0.0001
Gamma	1	68/158	43.0 (35.3, 50.8)	<0.05	-	
Expectoration	5	80/998	7.5 (2.9, 12.1)	<0.05	94.8%	0.012
Nasal congestion	7	105/6011	3.4 (1.8, 5.1)	<0.05	93.2%	0.0409
Sore throat	7	1045/18159	4.3 (1.3, 7.4)	<0.05	99.4%	0.0287
mMRC = 0	6	799/1317	50.6 (28.0, 73.2)	<0.05	98.9%	0.0187
mMRC≥1	8	590/1510	46.2 (28.1, 64.2)	<0.05	98.5%	0.114
**Cardiovascular symptoms**						
Short Breath	8	3848/13202	23.1 (12.1, 34.0)	<0.05	99.2%	0.1063
Palpitations	13	285/9764	3.8 (2.6, 5.0)	<0.05	93.8%	<0.0001
**Gastrointestinal symptoms**						
≥1 symptoms	5	35/1603	1.8 (0.2, 3.3)	<0.05	84.3%	<0.0001
Loss of appetite	8	107/2710	3.6 (1.8, 5.3)	<0.05	84.7%	<0.0001
Nausea	6	520/12225	3.6 (1.1, 6.0)	<0.05	95.7%	<0.0001
Diarrhea	10	121/3222	2.6 (1.3, 3.9)	<0.05	90.9%	0.0292
Abdominal pain	9	694/18327	2.6 (0.7, 4.5)	<0.05	98.7%	0.1204
Constipation	5	99/2969	4.1 (2.0, 6.2)	<0.05	92.4%	<0.0001
**Neurological and psychiatric symptoms**						
≥1 Neurological symptoms	8	371/2950	13.8 (8.5, 19.2)	<0.05	95.7%	0.0037

Paresthesias	9	1504/14363	11.4 (6.5, 16.4)	<0.05	99.2%	<0.0001
Memory problem	7	2019/12663	16.8 (10.3, 23.3)	<0.05	97.8%	0.005
Sleep difficulty	18	3758/21121	21.8 (16.2, 27.4)	<0.05	99.6%	<0.0001
Depression	11	2686/14896	17.9 (10.3, 25.5)	<0.05	99.4%	0.4603
Anxiety	13	1639/14470	13.5 (9.5, 17.4)	<0.05	97.8%	0.0792
Difficulty concentrating	8	3912/18093	22.3 (8.8, 35.8)	<0.05	99.9%	0.0094
**PFT**						
FEV1<80%	5	70/425	16.5 (9.0, 24.0)	<0.05	78.7%	0.0552
TLC<80%	6	204/828	27.0 (13.1, 41.0)	<0.05	96.4%	0.0961
DLCO<80%	6	866/1413	56.9 (40.6, 73.1)	<0.05	96.3%	0.7422
**CT results**						
CT abnormalities	11	1540/2206	60.5 (40.4, 80.6)	<0.05	99.2%	0.4432
GGO	12	403/1231	38.9 (26.8, 51.0)	<0.05	95.7%	0.1506
Consolidation	5	21/388	5.4 (0.8, 9.9)	<0.05	80.6%	0.0002
Fibrosis	9	340/1790	24.4 (13.3, 35.4)	<0.05	96.3%	0.8744
Bronchiectasis	7	195/892	22.2 (9.7, 34.7)	<0.05	96.6%	0.0136
**EQ-5D-5L**						
Mobility	5	192/1038	24.0 (12.9, 35.1)	<0.05	95.7%	0.0159
Personal care	4	73/911	14.8 (7.0, 22.5)	<0.05	97.8%	<0.0001
Usual activity	4	185/796	33.5 (13.1, 53.9)	<0.05	97.6%	0.0015
Pain or discomfort	5	430/1039	48.6 (35.4, 61.7)	<0.05	94.6%	<0.0001
Anxiety and depression	5	346/1014	36.7 (14.9, 58.5)	<0.05	98.9%	<0.0001

Notes: mMRC: Modified Medical Research Council Dyspnea Scale; PFT: Pulmonary functional test; FEV1: Forced expiratory volume in one second; TLC: Total lung capacity; DLCO: Carbon monoxide diffusing capacity; CT: Computerized tomography; GGO: Ground-glass opacity; EQ-5D-5L: Quality of life evaluation.

References

1. Emecen, A.N.; Keskin, S.; Turunc, O.; Suner, A.F.; Siyve, N.; Basoglu Sensoy, E.; Dinc, F.; Kilinc, O.; Avkan Oguz, V.; Bayrak, S., et al. The presence of symptoms within 6 months after COVID-19: a single-center longitudinal study. *Irish Journal of Medical Science* **2022**, 10.1007/s11845-022-03072-0, doi:10.1007/s11845-022-03072-0.
2. Och, A.; Tylicki, P.; Polewska, K.; Puchalska-Reglińska, E.; Parczewska, A.; Szabat, K.; Biedunkiewicz, B.; Dębska-ślizień, A.; Tylicki, L. Persistent post-covid-19 syndrome in hemodialyzed patients—a longitudinal cohort study from the North of Poland. *Journal of Clinical Medicine* **2021**, *10*, doi:10.3390/jcm10194451.
3. Lindahl, A.; Aro, M.; Reijula, J.; Mäkelä, M.J.; Ollgren, J.; Puolanne, M.; Järvinen, A.; Vasankari, T. Women report more symptoms and impaired quality of life: a survey of Finnish COVID-19 survivors. *Infectious Diseases* **2022**, *54*, 53-62, doi:10.1080/23744235.2021.1965210.
4. Todt, B.C.; Szlejf, C.; Duim, E.; Linhares, A.O.M.; Kogiso, D.; Varela, G.; Campos, B.A.; Baghelli Fonseca, C.M.; Polesso, L.E.; Bordon, I.N.S., et al. Clinical outcomes and quality of life of COVID-19 survivors: A follow-up of 3 months post hospital discharge. *Respiratory Medicine* **2021**, *184*, 106453, doi:https://doi.org/10.1016/j.rmed.2021.106453.
5. Becker, C.; Beck, K.; Zumbrunn, S.; Memma, V.; Herzog, N.; Bissmann, B.; Gross, S.; Loretz, N.; Mueller, J.; Amacher, S.A., et al. Long COVID 1 year after hospitalisation for COVID-19: a prospective bicentric cohort study. *Swiss Medical Weekly* **2021**, *151*, doi:10.4414/SMW.2021.W30091.
6. Dai, S.; Zhao, B.; Liu, D.; Zhou, Y.; Liu, Y.; Lan, L.; Li, Y.; Luo, W.; Zeng, Y.; Li, W. Follow-up study of the cardiopulmonary and psychological outcomes of covid-19 survivors six months after discharge in sichuan, china. *International Journal of General Medicine* **2021**, *14*, 7207-7217, doi:10.2147/IJGM.S337604.
7. Caruso, D.; Guido, G.; Zerunian, M.; Polidori, T.; Lucertini, E.; Pucciarelli, F.; Polici, M.; Rucci, C.; Bracci, B.; Nicolai, M., et al. Post-acute sequelae of COVID-19 pneumonia: Six-month chest CT follow-up. *Radiology* **2021**, *301*, E36-E405, doi:10.1148/radiol.2021210834.
8. Bretas, D.C.; Leite, A.S.; Mancuzo, E.V.; Prata, T.A.; Andrade, B.H.; Oliveira, J.D.G.F.; Batista, A.P.; Machado-Coelho, G.L.L.; Augusto, V.M.; Marinho, C.C. Lung function six months after severe COVID-19: Does time, in fact, heal all wounds? *Brazilian Journal of Infectious Diseases* **2022**, *26*, doi:10.1016/j.bjid.2022.102352.
9. Noviello, D.; Costantino, A.; Muscatello, A.; Bandera, A.; Consonni, D.; Vecchi, M.; Basilisco, G. Functional Gastrointestinal And Somatoform Symptoms Five Months After Sars-Cov-2 Infection: A Controlled Cohort Study. *Digestive and Liver Disease* **2021**, *53*, S119-S120, doi:10.1016/S1590-8658(21)00530-2.
10. Buonsenso, D.; Munblit, D.; Pazukhina, E.; Ricchiuto, A.; Sinatti, D.; Zona, M.; De Matteis, A.; D'Ilario, F.; Gentili, C.; Lanni, R., et al. Post-COVID Condition in Adults and Children Living in the Same Household in Italy: A Prospective Cohort Study Using the ISARIC Global Follow-Up Protocol. *Frontiers in Pediatrics* **2022**, *10*, doi:10.3389/fped.2022.834875.
11. Zuschlag, D.; Grandt, D.; Custodis, F.; Braun, C.; Häuser, W. Spontaneously reported

persistent symptoms related to coronavirus disease 2019 one year after hospital discharge : A retrospective cohort single-center study. *Schmerz* **2022**, 10.1007/s00482-022-00626-0, 1-9, doi:10.1007/s00482-022-00626-0.

12. Menges, D.; Ballouz, T.; Anagnostopoulos, A.; Aschmann, H.E.; Domenghino, A.; Fehr, J.S.; Puhan, M.A. Burden of post-COVID-19 syndrome and implications for healthcare service planning: A population-based cohort study. *PLoS ONE* **2021**, *16*, doi:10.1371/journal.pone.0254523.
13. Tessitore, E.; Handgraaf, S.; Poncet, A.; Achard, M.; Höfer, S.; Carballo, S.; Marti, C.; Follonier, C.; Girardin, F.; Mach, F., et al. Symptoms and quality of life at 1-year follow up of patients discharged after an acute COVID-19 episode. *Swiss Medical Weekly* **2021**, *151*, doi:10.4414/SMW.2021.W30093.
14. Zhou, F.; Tao, M.; Shang, L.; Liu, Y.; Pan, G.; Jin, Y.; Wang, L.; Hu, S.; Li, J.; Zhang, M., et al. Assessment of Sequelae of COVID-19 Nearly 1 Year After Diagnosis. *Frontiers in Medicine* **2021**, *8*, doi:10.3389/fmed.2021.717194.
15. Desgranges, F.; Tadini, E.; Munting, A.; Regina, J.; Filippidis, P.; Viala, B.; Karachalias, E.; Suttels, V.; Haefliger, D.; Kampouri, E., et al. Post‑COVID‑19 Syndrome in Outpatients: a Cohort Study. *Journal of General Internal Medicine* **2022**, *37*, 1943-1952, doi:10.1007/s11606-021-07242-1.
16. Förster, C.; Colombo, M.G.; Wetzel, A.J.; Martus, P.; Joos, S. Persisting Symptoms after COVID-19: Prevalence and Risk Factors in a Population-Based Cohort. *Deutsches Arzteblatt International* **2022**, *119*, 167-174, doi:10.3238/arztebl.m2022.0147.
17. Nesan, G.S.C.Q.; Keerthana, D.; Yamini, R.; Jain, T.; Kumar, D.; Eashwer, A.; Maiya, G.R. 3-Month Symptom-Based Ambidirectional Follow-up Study Among Recovered COVID-19 Patients from a Tertiary Care Hospital Using Telehealth in Chennai, India. *Inquiry : a journal of medical care organization, provision and financing* **2021**, *58*, 469580211060165, doi:10.1177/00469580211060165.
18. Prestes, G.D.S.; Simon, C.S.; Walz, R.; Ritter, C.; Dal-Pizzol, F. Respiratory Outcomes After 6 Months of Hospital Discharge in Patients Affected by COVID-19: A Prospective Cohort. *Frontiers in Medicine* **2022**, *9*, doi:10.3389/fmed.2022.795074.
19. Calcaianu, G.; Degoul, S.; Michau, B.; Payen, T.; Gschwend, A.; Fore, M.; Iamandi, C.; Morel, H.; Oster, J.-P.; Bizieux, A., et al. Mid-term pulmonary sequelae after hospitalisation for COVID-19: the French SISCOVID cohort. *Respiratory Medicine and Research* **2022**, https://doi.org/10.1016/j.resmer.2022.100933, 100933, doi:https://doi.org/10.1016/j.resmer.2022.100933.
20. Vargas Centanaro, G.; Calle Rubio, M.; Álvarez-Sala Walther, J.L.; Martinez-Sagasti, F.; Albuja Hidalgo, A.; Herranz Hernández, R.; Rodríguez Hermosa, J.L. Long-term Outcomes and Recovery of Patients who Survived COVID-19: LUNG INJURY COVID-19 Study. *Open Forum Infectious Diseases* **2022**, *9*, doi:10.1093/ofid/ofac098.
21. Labarca, G.; Henríquez-Beltrán, M.; Lastra, J.; Enos, D.; Llerena, F.; Cigarroa, I.; Lamperti, L.; Ormazabal, V.; Ramirez, C.; Espejo, E., et al. Analysis of clinical symptoms, radiological changes and pulmonary function data 4 months after COVID-19. *Clinical Respiratory Journal* **2021**, *15*, 992-1002, doi:10.1111/crj.13403.
22. Klein, H.; Asseo, K.; Karni, N.; Benjamini, Y.; Nir-Paz, R.; Muszkat, M.; Israel, S.; Niv, M.Y. Onset, duration and unresolved symptoms, including smell and taste changes, in mild

COVID-19 infection: a cohort study in Israeli patients. *Clinical Microbiology and Infection* **2021**, *27*, 769-774, doi:https://doi.org/10.1016/j.cmi.2021.02.008.

23. Martin-Loeches, I.; Motos, A.; Menéndez, R.; Gabarrús, A.; González, J.; Fernández-Barat, L.; Ceccato, A.; Pérez-Arnal, R.; García-Gasulla, D.; Ferrer, R., et al. ICU-Acquired Pneumonia Is Associated with Poor Health Post-COVID-19 Syndrome. *Journal of Clinical Medicine* **2022**, *11*, doi:10.3390/jcm11010224.
24. González, J.; Benítez, I.D.; Carmona, P.; Santisteve, S.; Monge, A.; Moncusí-Moix, A.; Gort-Paniello, C.; Pinilla, L.; Carratalá, A.; Zuil, M., et al. Pulmonary Function and Radiologic Features in Survivors of Critical COVID-19: A 3-Month Prospective Cohort. *Chest* **2021**, *160*, 187-198, doi:10.1016/j.chest.2021.02.062.
25. Aranda, J.; Oriol, I.; Martín, M.; Feria, L.; Vázquez, N.; Rhyman, N.; Vall-Llosera, E.; Pallarés, N.; Coloma, A.; Pestaña, M., et al. Long-term impact of COVID-19 associated acute respiratory distress syndrome. *Journal of Infection* **2021**, *83*, 581-588, doi:10.1016/j.jinf.2021.08.018.
26. Zhang, J.; Shu, T.; Zhu, R.; Yang, F.; Zhang, B.; Lai, X. The Long-Term Effect of COVID-19 Disease Severity on Risk of Diabetes Incidence and the Near 1-Year Follow-Up Outcomes among Postdischarge Patients in Wuhan. *Journal of Clinical Medicine* **2022**, *11*, doi:10.3390/jcm11113094.
27. Kingery, J.R.; Safford, M.M.; Martin, P.; Lau, J.D.; Rajan, M.; Wehmeyer, G.T.; Li, H.A.; Alshak, M.N.; Jabri, A.; Kofman, A., et al. Health Status, Persistent Symptoms, and Effort Intolerance One Year After Acute COVID-19 Infection. *Journal of General Internal Medicine* **2022**, *37*, 1218-1225, doi:10.1007/s11606-021-07379-z.
28. Zhan, K.; Zhang, X.; Wang, B.; Jiang, Z.; Fang, X.; Yang, S.; Jia, H.; Li, L.; Cao, G.; Zhang, K., et al. Short-and long-term prognosis of glycemic control in COVID-19 patients with type 2 diabetes. *QJM* **2022**, *115*, 131-139, doi:10.1093/qjmed/hcac020.
29. Miskowiak, K.W.; Fugledalen, L.; Jespersen, A.E.; Sattler, S.M.; Podlekareva, D.; Rungby, J.; Porsberg, C.M.; Johnsen, S. Trajectory of cognitive impairments over 1 year after COVID-19 hospitalisation: Pattern, severity, and functional implications. *European Neuropsychopharmacology* **2022**, *59*, 82-92, doi:10.1016/j.euroneuro.2022.04.004.
30. Sigfrid, L.; Drake, T.M.; Pauley, E.; Jesudason, E.C.; Olliaro, P.; Lim, W.S.; Gillesen, A.; Berry, C.; Lowe, D.J.; McPeake, J., et al. Long Covid in adults discharged from UK hospitals after Covid-19: A prospective, multicentre cohort study using the ISARIC WHO Clinical Characterisation Protocol. *The Lancet Regional Health - Europe* **2021**, *8*, 100186, doi:https://doi.org/10.1016/j.lanepe.2021.100186.
31. Peghin, M.; Palese, A.; Venturini, M.; De Martino, M.; Gerussi, V.; Graziano, E.; Bontempo, G.; Marrella, F.; Tommasini, A.; Fabris, M., et al. Post-COVID-19 symptoms 6 months after acute infection among hospitalized and non-hospitalized patients. *Clinical Microbiology and Infection* **2021**, *27*, 1507-1513, doi:10.1016/j.cmi.2021.05.033.
32. Colizzi, M.; Peghin, M.; De Martino, M.; Bontempo, G.; Gerussi, V.; Palese, A.; Isola, M.; Tascini, C.; Balestrieri, M. Mental health symptoms one year after acute COVID-19 infection: Prevalence and risk factors. *Revista de Psiquiatría y Salud Mental* **2022**, https://doi.org/10.1016/j.rpsm.2022.05.008, doi:https://doi.org/10.1016/j.rpsm.2022.05.008.
33. Vejen, M.; Hansen, E.F.; Al-Jarah, B.N.I.; Jensen, C.; Thaning, P.; Jeschke, K.N.; Ulrik, C.S.

Hospital admission for COVID-19 pneumonitis - long-term impairment in quality of life and lung function. *Eur Clin Respir J* **2022**, *9*, 2024735, doi:10.1080/20018525.2021.2024735.
34. Aiello, M.; Marchi, L.; Calzetta, L.; Speroni, S.; Frizzelli, A.; Ghirardini, M.; Celiberti, V.; Sverzellati, N.; Majori, M.; Mori, P.A., et al. Coronavirus Disease 2019: COSeSco - A Risk Assessment Score to Predict the Risk of Pulmonary Sequelae in COVID-19 Patients. *Respiration* **2021**, 10.1159/000519385, doi:10.1159/000519385.
35. Rivera-Izquierdo, M.; Láinez-Ramos-Bossini, A.J.; de Alba, I.G.; Ortiz-González-Serna, R.; Serrano-Ortiz, Á.; Fernández-Martínez, N.F.; Ruiz-Montero, R.; Cervilla, J.A. Long COVID 12 months after discharge: persistent symptoms in patients hospitalised due to COVID-19 and patients hospitalised due to other causes-a multicentre cohort study. *BMC Med* **2022**, *20*, 92, doi:10.1186/s12916-022-02292-6.
36. Rigoni, M.; Torri, E.; Nollo, G.; Donne, L.D.; Rizzardo, S.; Lenzi, L.; Falzone, A.; Cozzio, S. "Long COVID" results after hospitalization for SARS-CoV-2 infection. *Scientific reports* **2022**, *12*, 9581, doi:10.1038/s41598-022-13077-5.
37. Mølhave, M.; Leth, S.; Gunst, J.D.; Jensen-Fangel, S.; Østergaard, L.; Wejse, C.; Agergaard, J. Long-term symptoms among hospitalized COVID-19 patients 48 weeks after discharge—A prospective cohort study. *Journal of Clinical Medicine* **2021**, *10*, doi:10.3390/jcm10225298.
38. Porto, M.H.; Delgado, T.; Aguirre-Jaime, A.; Ramos, M.J.; Campos, S.; Acosta, O.; Llanos, A.B.; Lecuona, M. Patients at risk of pulmonary fibrosis Post Covid-19: Epidemiology, pulmonary sequelaes and humoral response. 2022; 10.1101/2022.03.04.22271920.
39. Attauabi, M.; Dahlerup, J.F.; Poulsen, A.; Hansen, M.R.; Vester-Andersen, M.K.; Eraslan, S.; Prahm, A.P.; Pedersen, N.; Larsen, L.; Jess, T., et al. Outcomes and Long-Term Effects of COVID-19 in Patients with Inflammatory Bowel Diseases - A Danish Prospective Population-Based Cohort Study with Individual-Level Data. *Journal of Crohn's and Colitis* **2022**, *16*, 757-767, doi:10.1093/ecco-jcc/jjab192.
40. Hossain, M.A.; Hossain, K.M.A.; Saunders, K.; Uddin, Z.; Walton, L.M.; Raigangar, V.; Sakel, M.; Shafin, R.; Hossain, M.S.; Kabir, M.F., et al. Prevalence of Long COVID symptoms in Bangladesh: A prospective Inception Cohort Study of COVID-19 survivors. *BMJ Global Health* **2021**, *6*, doi:10.1136/bmjgh-2021-006838.
41. Eloy, P.; Tardivon, C.; Martin-Blondel, G.; Isnard, M.; Turnier, P.L.; Marechal, M.L.; CabiÉ, A.; Launay, O.; Tattevin, P.; Senneville, E., et al. Severity of self-reported symptoms and psychological burden 6-months after hospital admission for COVID-19: a prospective cohort study. *International Journal of Infectious Diseases* **2021**, *112*, 247-253, doi:10.1016/j.ijid.2021.09.011.
42. Duggal, P.; Penson, T.; Manley, H.N.; Vergara, C.; Munday, R.M.; Duchen, D.; Linton, E.A.; Zurn, A.; Keruly, J.C.; Mehta, S.H., et al. Post-sequelae symptoms and comorbidities after COVID-19. *Journal of Medical Virology* **2022**, *94*, 2060-2066, doi:10.1002/jmv.27586.
43. Mónica, R.-S.; Maribel, Q.-F.; Javier, J.; Isabel, L.-M.; Rocío, T.; Rocío, A.; Javier, G.-P.F. Cardiac complications in a geriatric population hospitalized with COVID-19: The OCTA-COVID cohort. *Revista Española de Geriatría y Gerontología* **2022**, *57*, 63-70, doi:https://doi.org/10.1016/j.regg.2022.01.003.
44. Peter, R.S.; Nieters, A.; Kräusslich, H.G.; Brockmann, S.O.; Göpel, S.; Kindle, G.; Merle, U.;

Steinacker, J.M.; Rothenbacher, D.; Kern, W.V., et al. Prevalence, determinants, and impact on general health and working capacity of post-acute sequelae of COVID-19 six to 12 months after infection: a population-based retrospective cohort study from southern Germany. 2022; 10.1101/2022.03.14.22272316.

45. Robey, R.C.; Kemp, K.; Hayton, P.; Mudawi, D.; Wang, R.; Greaves, M.; Yioe, V.; Rivera-Ortega, P.; Avram, C.; Chaudhuri, N. Pulmonary Sequelae at 4 Months After COVID-19 Infection: A Single-Centre Experience of a COVID Follow-Up Service. *Advances in Therapy* **2021**, *38*, 4505-4519, doi:10.1007/s12325-021-01833-4.
46. Titze-de-Almeida, R.; da Cunha, T.R.; dos Santos Silva, L.D.; Ferreira, C.S.; Silva, C.P.; Ribeiro, A.P.; de Castro Moreira Santos Júnior, A.; de Paula Brandão, P.R.; Silva, A.P.B.; da Rocha, M.C.O., et al. Persistent, new-onset symptoms and mental health complaints in Long COVID in a Brazilian cohort of non-hospitalized patients. *BMC Infectious Diseases* **2022**, *22*, doi:10.1186/s12879-022-07065-3.
47. Zayet, S.; Zahra, H.; Royer, P.Y.; Tipirdamaz, C.; Mercier, J.; Gendrin, V.; Lepiller, Q.; Marty-Quinternet, S.; Osman, M.; Belfeki, N., et al. Post-COVID-19 Syndrome: Nine Months after SARS-CoV-2 Infection in a Cohort of 354 Patients: Data from the First Wave of COVID-19 in Nord Franche-Comté Hospital, France. *Microorganisms* **2021**, *9*, doi:10.3390/microorganisms9081719.
48. Liao, X.; Li, D.; Liu, Z.; Ma, Z.; Zhang, L.; Dong, J.; Shi, Y.; Gu, X.; Zheng, G.; Huang, L., et al. Pulmonary Sequelae in Patients After Recovery From Coronavirus Disease 2019: A Follow-Up Study With Chest CT. *Frontiers in Medicine* **2021**, *8*, doi:10.3389/fmed.2021.686878.
49. Li, Y.; Han, X.; Huang, J.; Alwalid, O.; Jia, X.; Yuan, M.; Cao, Y.; Shao, G.; Cui, Y.; Liu, J.; et al. Follow-up study of pulmonary sequelae in discharged COVID-19 patients with diabetes or secondary hyperglycemia. Eur. J. Radiol. **2021**, 144, 109997. https://doi.org/10.1016/j.ejrad.2021.109997..
50. Aparisi, Á.; Ybarra-Falcón, C.; García-Gómez, M.; Tobar, J.; Iglesias-Echeverría, C.; Jaurrieta-Largo, S.; Ladrón, R.; Uribarri, A.; Catalá, P.; Hinojosa, W., et al. Exercise Ventilatory Inefficiency in Post-COVID-19 Syndrome: Insights from a Prospective Evaluation. *J Clin Med* **2021**, *10*, doi:10.3390/jcm10122591.
51. Romero-Duarte, Á.; Rivera-Izquierdo, M.; Guerrero-Fernández de Alba, I.; Pérez-Contreras, M.; Fernández-Martínez, N.F.; Ruiz-Montero, R.; Serrano-Ortiz, Á.; González-Serna, R.O.; Salcedo-Leal, I.; Jiménez-Mejías, E., et al. Sequelae, persistent symptomatology and outcomes after COVID-19 hospitalization: the ANCOHVID multicentre 6-month follow-up study. *BMC Medicine* **2021**, *19*, doi:10.1186/s12916-021-02003-7.
